# Isotopic and Elemental Fingerprint of Edible Egg Parts—The Health Risk Assessment Based on Potentially Toxic Elements Content

**DOI:** 10.3390/molecules28020503

**Published:** 2023-01-04

**Authors:** Gabriela Cristea, Adriana Dehelean, Romulus Puscas, Ariana Raluca Hategan, Dana Alina Magdas

**Affiliations:** National Institute for Research and Development of Isotopic and Molecular Technologies, 67-103 Donat Street, 400293 Cluj-Napoca, Romania

**Keywords:** egg, isotopic fingerprint, elemental content, rearing system, health risk assessment

## Abstract

The present study investigated the isotopic and elemental profile (by IRMS and ICP–MS) of edible egg parts (29 egg whites and 29 yolks) mainly collected from Romania. In order to differentiate the egg white and yolk coming from different hen rearing systems (backyard and barn), Partial Least Square-Discriminant Analysis (PLS-DA) models were developed. The models’ accuracies for the discrimination according to the hen growing system were 96% for egg white and 100% for egg yolk samples, respectively. Elements that proved to have the highest discrimination power for both egg white and yolk were the following: δ^13^C, Li, B, Mg, K, Ca, Mn, Fe, Co, Zn, Rb, Sr, Mo, Ba, La, Ce, and Pb. Nevertheless, the important compositional differentiation, in terms of essential mineral content, between the edible egg parts (egg white and egg yolk) were also pointed out. The estimated daily intake (EDI), the target hazard quotient (THQ) for Cr, Mn, Fe, Co, Ni, Cu, Zn, Se, Cd, Pb, and As, as well as the hazard index (HI) were used to assess non-carcinogenic human health risks from egg consumption. The obtained results showed no noticeable health risks related to egg consumption for humans from the point of view of the potentially toxic metals.

## 1. Introduction

The egg is one of the staple foods, due to its nutritional and biological value, being a source of protein, fat, and minerals [1]. In the last decade, from 2008 to 2018, global egg production experienced a spectacular increase of 24% [2] and it is predicted to have an ongoing growth of around 3% per year. Mexico has the highest consumption per capita, reaching an average of 355 eggs per person per year, followed by China (344) and Japan (325) [3]. At the European Union level, in 2019, France had the largest production of eggs, 13.100 million, followed by Spain (12.871 million), and Poland (10.291 million). Romania registered 4.887 million eggs [4].

In this period of economic crisis, since the war started in Eastern Europe, there has been an estimated increase in egg consumption, in the context of declining purchasing power. Eggs are a low-cost source of protein, cheaper than other types of protein, and can be prepared and served in many ways. The concept of quality associated with the origin of the product and the way the chickens were raised is a concern. The concept is one found in several categories of food products, favoring the natural, traditional character of the product. Isotopic fingerprinting represents a recognized technique in forensic research in assigning the geographic origin and production method (organic versus conventional) of food items, or the rearing system of animals [5]. The isotopic signature of animal products is influenced by the isotopic value of the water they drink and the plants they consume [6]. The corroboration of analytical results obtained by Isotope Ratios Mass Spectrometry (IRMS) and Inductively Coupled Plasma–Mass Spectrometry (ICP–MS), followed by statistical data treatment, could lead to precious data for tracing agricultural products’ geographical origin, and the relevant animal’s diet and growing regime [7,8,9].

A major worldwide concern is food safety, because ingestion of food items is a pathway for human exposure to unsafe food ingredients. It has been demonstrated that eggs are capable of accumulating metals through the feeding of poultry as well as the environment in which they are reared [10], and metals are indicator for estimating human health risk through their consumption. Some metals, such as zinc, lead, selenium, manganese, iron, and cobalt, represent essential elements and they need to be ingested at adequate levels to maintain physiological functions [11]. However, when the intake of these metals is exceeded for a long time, adverse effects on human health arise. On the other hand, there are well-documented studies that indicate that nonessential elements, such as lead, arsenic, cadmium and nickel, have adverse health effects even at low levels [12,13].

In this context, the calculation of the heavy metal risk factors for consumers is of great importance. The daily consumption of a food item, and the concentrations obtained for each metal, as well as body weight, represent the parameters taken into account to calculate EDI value. THQ is defined as the ratio of exposure to the toxic element and the reference dose which is the highest level at which no adverse health effects are expected. HI is used to estimate the total non-carcinogenic health risks considering all the studied metals assuming dose additivity. PTDI represents the maximum daily exposure level to a contaminant. Thus, a comparison of the estimated daily intake (EDI) with the provisional tolerable daily intake (PTDI), the target hazard quotient (THQ), and the hazard index (HI) recommended by international food laws and regulatory agencies could be valuable to evaluate the potential health hazard by food item consumption. Different studies have been reported based on the evaluation of the potential human health risk associated with consumption of foodstuffs (e.g., meat, milk, seafood, fish, egg) [14,15,16,17]. To our knowledge, there has not been any study on this research area in Romania.

The aim of the present study was to develop PLS–DA models in order to differentiate the edible egg parts (egg white and yolk) coming from two hen rearing systems (backyard and barn), considering both isotopic patterns and the elemental composition of the egg constituents. The variables with the highest differentiation potential were identified. Furthermore, in this study we aimed to assess the risk to human health associated with heavy metal intake due to the consumption of eggs. In this regard, THQ and HI were used to assess non-carcinogenic human health risks.

## 2. Results and Discussion

The egg white and yolk samples coming from different hen rearing systems (backyard and barn) were investigated from the points of view of isotopic (δ^13^C) and multi-elemental (Li, B, Na, Mg, K, Ca, Sc, Ti, V, Cr, Mn, Fe, Co, Ni, Cu, Zn, As, Se, Rb, Sr, Zr, Nb, Mo, Pd, Ag, Cd, Sn, Sb, Ba, La, Ce, Pr, Gd, Pt and Pb) compositions The model-based feature selection method was applied, based on the entire set of isotope and elemental determinations (i.e., 36 variables) in order to determine the parameters having the highest classification power, namely 17 variables (δ^13^C, Li, B, Mg, K, Ca, Sc, Ti, V, Cr, Mn, Fe, Co, Ni, Zn, As, Rb, Sr, Zr, Mo, Ag, Sn, Sb, Ba, La, Ce, Pr, Gd and Pb). Table 1 shows the minimum and maximum values of the parameters. Details regarding the discrimination models of samples, according to the rearing system, are presented in the following paragraphs.

### 2.1. Isotopic Fingerprint of Egg Samples

The ^13^C isotopic signature of plants differs as a function of the photosynthetic cycle, C3 or C4. In the C3 cycle (or Calvin), the initial chemical product formed during the carboxylation reaction in the majority of plant families (vegetables, fruits, and cereals) is a three-carbon molecule. The enzyme involved in the carboxylation reaction is Rubisco. C3 plants have δ^13^C values in the range of −30‰ to −23‰ (e.g., wheat, barley, oats, sunflower, etc.) [18]. In the C4 cycle (or Hatch–Slack), carboxylation by another enzyme, PEP (phospfoenolpyruvate carboxylase) yields a dicarboxylic acid with four carbon atoms. Maize and sugar cane are the most known examples of C4 plants. The δ^13^C values for C4 plants have a different isotopic signature, varying between −14‰ and −12‰. Thus, the ^13^C isotopic values of the analyzed egg samples reflected the proportion of C3 and C4 plants introduced into the hen’s diet.

The range of variation for δ^13^C values of egg white was between −25.0 and −14.0‰ for samples coming from the backyard rearing system (mean value of −19.8‰), and from −21.8 to −15.9‰ (mean value of −19.1‰) for samples originating from industrial farms. Regarding egg yolk, the ^13^C isotopic fingerprints varied from −25.3 to −14.4‰ for samples obtained from rural regions, and between −23.7 and −15.9‰ for those from the industrial growing system. The obtained data showed that, in industrial farms, a combined diet, C3 and C4, was used, while hens from rural regions were fed by a higher proportion of corn in the central part of Romania, this feeding being a tradition not only for hens’ diet, but also for swine feeding regimes [8]. Two samples from the backyard rearing system, from the south-east part of Romania, proved a feeding regime exclusively based on C3 plants, having the most depleted ^13^C values of yolk (−25.3‰). Our results fit those published by Rogers (2009) [9]. For example, his results for delipidated yolk from free range and organic eggs had δ^13^C values between −24.8 and −16.3‰, and from −24.7 to −18.1‰ for caged and barn eggs.

**Figure 1:**
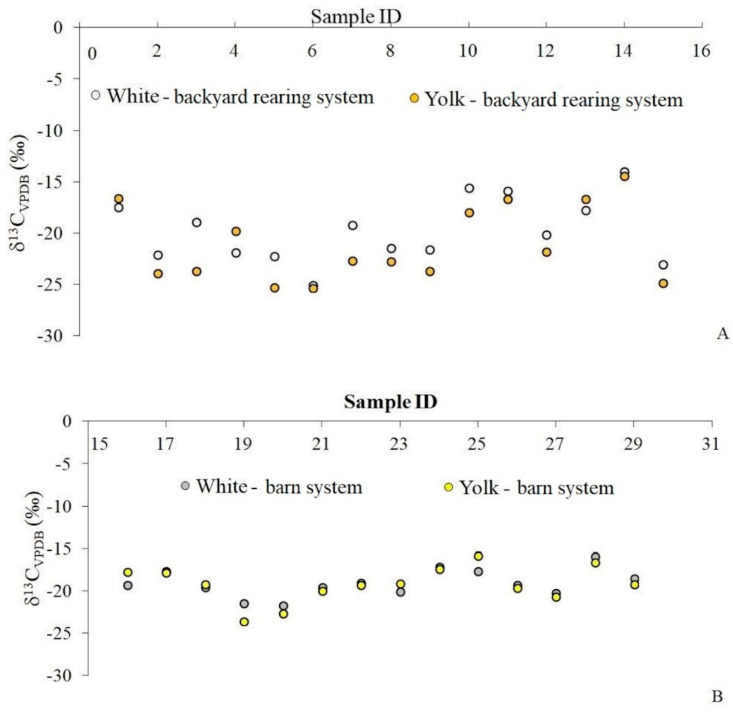
The δ^13^C values for investigated egg samples coming from: (**A**) backyard rearing system; (**B**) barn system.

As can be observed in Figure 1A,B, there is a higher difference among isotopic values of egg white–yolk pairs of samples coming from backyard reared chickens (maximum value 4.7‰), as compared to those of eggs originating from industrial farms (maximum value 2.2‰). The production of an egg is a gradual process over a period of 25–26 h. The yolk begins its journey 10 days before it is encompassed in a shell [19]. Many organs contribute to the conversion of the raw materials from the feed eaten by hens into the nutrients and substances that become part of the egg [9]. Most of the albumen formation takes place in the magnum, and this process lasts about 3 to 4 h [20]. In this context, the obtained isotopic values could be explained by the fact that poultry that are free-reared have access to foraging in soil, and pecking seeds, insects, worms, and grasses, in addition to their feed given by breeders. In our country, this system of rearing is perceived by consumers as one that leads to a better taste of chicken meat, and subsequently of eggs. As yolk and egg white are formed at different time moments, and the backyard laying hens’ diet varies from one day to another, differences in the isotopic fingerprint of ^13^C for egg white and yolk appear, reflecting the different signatures of feed. Chickens from a barn system are fed by concentrates containing cereals (wheat, barley, soybeans, corn), minerals, vitamins, calcium carbonate, and salt. In this growing system, the diet is the same for laying hens of the same age, not changing from one day to another, and this fact is reflected in the isotopic values of egg white and yolk, with these values being closer.

### 2.2. Content of Macro-Elements, Micro-Elements, and Trace Elements in Egg White and Yolk Samples

A total of 35 elements, namely, macro-elements (K, Na, Mg and Ca), micro-elements (Fe, Zn, Rb, Cu, Cr, B, Ni, Ba, Mn, Se, Li, Sc, Ti, V, Pd, Sr), and trace elements (Co, La, Nb, Ce, Pr, Gd, Zr, Mo, Ag, Pt, Pb, Sn, Sb, As, Cd) were determined from the egg white and yolk samples (Table 2).

It is well known that some of these elements, like Mn, Co, Cr, Fe, Se, Zn, and Cu, are essential in low concentrations for human health but can have a toxic nature if present above certain concentrations. In order to ensure that potentially toxic trace elements do not pose a risk to human health, institutions, such as the World Health Organization (WHO) [21] and European Commission by European Food Safety Authority (EFSA) [22], have developed risk analysis as a tool for strengthening food safety systems and for reducing diseases linked to food consumption. In the present study, the health risk assessment of potentially toxic elements, particularly Cr, Mn, Fe, Co, Ni, Cu, Zn, Se, Cd, Pb and As, are studied and presented.

#### 2.2.1. The Main Macro-Elements, Micro-Elements, and Trace Elements (in mg/kg Fresh Sample) in Egg White

The average content (in mg/kg fresh sample) of Na, K, Mg and Ca in samples of eggs from hens reared in backyard rearing systems were 1748.27; 838.00; 108.35; 49.67 and those from barn systems were 1812.17; 798.18; 116.22 and 42.40. The mean Fe, Zn, Cu, Cr, Mn Se, Li and B levels in albumen samples coming from eggs of hens in the two rearing systems were: 2.98 (backyard) and 1.36 (barn system); 0.18 (backyard) and 0.08 (barn system); 0.19 (backyard) and 0.20 (barn); 0.35 (backyard) and 0.42 (barn); 0.02 (backyard) and 0.01 (barn); 0.10 (for both rearing system of hen); 0.31 (backyard) and 0.01 (barn), 0.24 (backyard) and 0.30 (barn). In the case of the potentially toxic elements, the mean concentration in samples of the eggs coming from backyard-reared hens were 0.05 (Pb), 0.03 (Sn), 0.0005 (Sb), 0.02 (As), 0.001 (Cd). For the samples from hens reared in the barn system, the average levels of these elements were found to be 0.01 (Pb), 0.04 (Sn), 0.0004 (Sb), 0.03 (As) and 0.001 (Cd). The mean concentration of Na, Mg, Cu, Cr, B, Sn and As in the egg white was higher in samples coming from barns than from samples coming from backyards. On the other hand, the mean concentration in egg white samples coming from the backyard rearing system was higher than that for egg white samples from the barn system for K, Ca, Li, Fe, Zn, Mn, Pb and Sb.

#### 2.2.2. The Main Macro-Elements, Micro-Elements and Trace Elements (in mg/kg Fresh Sample) in Egg Yolk

The mean values of macro-elements (in mg/kg fresh sample) were the following: 887.12 for Na, 1350.70 for Ca, 1292.95 for K and 180.08 for Mg in samples coming from backyard-reared hens; 830.47 for Na, 1364.54 for Ca, 1028.51 for K and 164.24 for Mg for barn system, respectively. The average contents of micro-elements (Fe, Zn, Cu, Cr, Mn, Se, Li and B) were 114.27; 30.6; 2.40; 3.28; 1.34; 0.60; 0.11 and 0.22 in samples from the eggs coming from the backyard rearing system; 94.06; 28.00; 2.35; 3.16; 1.21; 0.56; 0.01 and 0.33 in samples originating from the barn system. The mean trace elements concentrations (Co, Mo and Pt) were 0.02; 0.08; 0.02 and 0.01; 0.12; 0.03, respectively, for yolk samples coming from the two rearing systems (backyard and barn, respectively). The mean levels of Pb, Sn, Sb, As and Cd in the samples from backyard reared hens were 0.06; 0.23; 0.002; 0.05; 0.005 and 0.03; 0.31; 0.002; 0.05 and 0.01 for the yolk samples originating from the barn system.

The mean concentrations of Na, Mg, K, Li, Fe, Zn, Cu, Cr, Mn, Se, Pb and Sb in the egg yolk were higher in samples coming from backyard-reared hens than from samples coming from the barn system. The mean concentration in yolk samples coming from the barn system was higher than for yolk samples from the backyard rearing system for Ca, B, Sn and Cd.

Therefore, the yolk was the main storage compartment for different elements (Mg, K, Ca, Fe, Cu, Zn, Se, Cr and Mn) in the eggs. The egg white was the main source of Na and contained low levels of metals, such as Cu, Fe, Zn, Cr and Mn.

### 2.3. Developing of PLS–DA Models

For our study, the supervised statistic treatment was performed using Partial Least Squares Discriminant Analysis (PLS–DA).

#### 2.3.1. The Classification According to Hen’s Growing System—Egg White Samples

The first category of PLS–DA differentiation models aimed to classify the egg samples in terms of the animal growing system, based on the isotope and elemental concentrations determined by analyzing the egg white. In this regard, the experimental data associated with 15 samples collected from backyard rearing systems and 14 samples from barn systems were utilized for the development and validation of the classification models.

When the PLS–DA model was constructed, based on the entire set of isotope and elemental determinations (i.e., 36 variables), an accuracy of 86% was obtained in the cross-validation procedure. This result corresponded to the use of the information associated with the first 3 latent variables (LVs), a case in which the cross-validation classification error average reached the lowest value. Two egg white samples collected from each type of growing system were misclassified by the PLS–DA model, leading to 85% and 86% true positive rates with respect to the barn and backyard rearing system classes, respectively.

**Figure 2:**
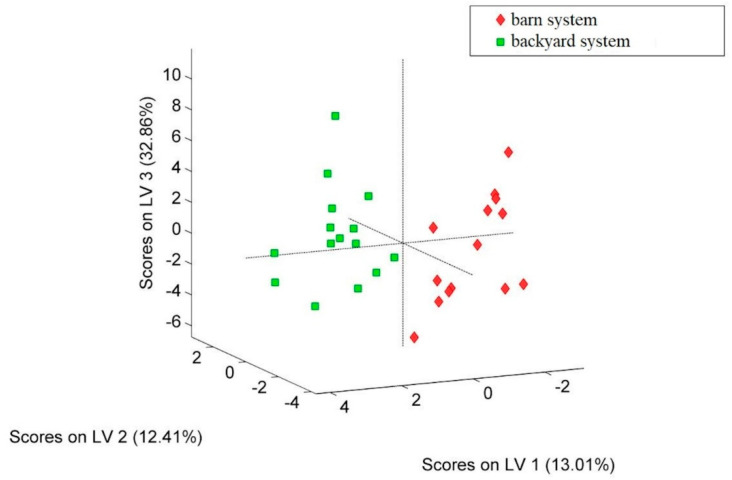
PLS–DA score plot associated with the differentiation of the egg white samples in terms of the hen growing system.

When the model-based feature selection method was applied, in order to determine the most powerful discriminators for the egg white samples, the following were obtained: δ^13^C, Li, B, Mg, K, Ca, Sc, Ti, V, Cr, Mn, Fe, Co, Ni, Zn, As, Rb, Sr, Zr, Mo, Ag, Sn, Sb, Ba, La, Ce, Pr, Gd and Pb. The most significant features identified were used to develop a new PLS–DA model, based on these 29 variables (isotope and elemental content) (Figure 2).

The chosen number of LVs was 9, as it illustrated the lowest cross-validation classification error average. The model was able to correctly predict the growing regime for 28 samples, leading to a total accuracy score of 96%. Only one sample was wrongly classified, namely, one sample collected from a backyard rearing system was attributed to the barn system group.

#### 2.3.2. The Classification According to Hen’s Growing System—Egg Yolk Samples

The same data processing workflow as the one previously described was adopted for differentiating the egg yolk samples with respect to the rearing system. The number and the distribution of the samples in terms of the growing regime were the same as for the development of the egg white classification models, illustrating a data set consisting of 15 samples collected from backyard rearing systems and 14 from the barn system.

Firstly, the entire set of elemental and isotope determinations was used as input data for the construction of the PLS–DA model. The best classification performance corresponded to 93% accuracy and was obtained by setting the number of LVs to 2. In this case, one egg yolk sample from the barn system and another one from a backyard rearing system were wrongly predicted in the cross-validation procedure.

The variables that were identified as having the highest differentiation potential were: δ^13^C, Li, B, Mg, K, Ca, Mn, Fe, Co, Zn, Rb, Sr, Mo, Ba, La, Ce, Pb. An interesting aspect was reflected by the fact that all these elemental and isotope determinations were also found to be markers for the classification of the egg white samples.

**Figure 3:**
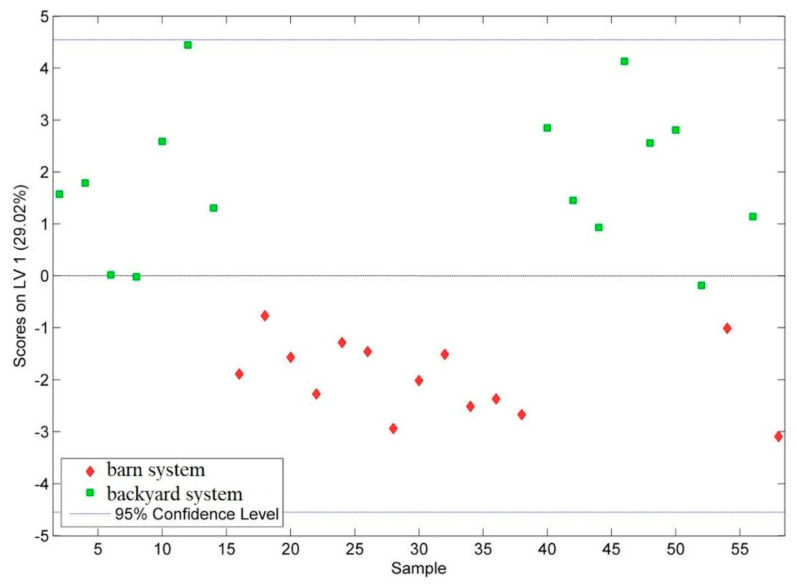
PLS–DA score plot associated to the classification of the egg yolk samples with respect to the hen growing system.

When the PLS–DA model was reconstructed, based on the information associated with these 17 features, a percentage of 100% for discrimination of the egg yolk samples in terms of the growing regime was achieved. The classifier was built by taking into account only the first computed LV, and the scores of the samples corresponding to this direction are presented in Figure 3.

Foods of animal origin are rich in naturally occurring micro-elemental lithium, such as milk, poultry meat and eggs (>7000 µg dry matter) [23]. As cereals constitute an important part in the poultry diet, and grains contain a low content of boron, 2 mg/kg of B are recommended for hens’ feed, even if B is not considered an essential micronutrient for fowls and other farm animals [24]. The laying hens’ diet must be enriched with vitamins, minerals, enzymes, and amino acids. In general premixes are used. An additive intended for laying hens must include the following: zinc (to support heart function), iron (to prevent anemia), and manganese (to prevent pathologies of the joints of the legs). In this context, it is not surprising to obtain these elements as principal markers of differentiation for laying hens’ rearing system.

### 2.4. Human Health Risk Assessment and Estimation of Non-Carcinogenic Risk

The essential trace elements and metals with toxic potential concentrations in whole egg are indicated in Table 3.

According to the reported data from the literature [4], a Romanian person eats, on average, 240–250 eggs per year, which represents consumption of 20 to 22 eggs in a month, or 0.66 to 0.73 eggs per day. In this study, the daily ingestion rate (IRd in g/day) was calculated to be 40 g and the body weight (BW) was taken as 70 kg (for an adult). In order to evaluate the safety of the investigated eggs, with respect to their heavy metal levels, the daily intake of metals was calculated from egg consumption and compared with the provisional tolerable daily intake (PTDI) for humans (Table 4).

Cr, Mn, Fe, Co, Ni, Cu, Zn, As, Se, Cd and Pb contents (μg/kg/body weight/day) in the EDI of eggs in the present study ranged from 0.526 to 1.178 (Cr), 0.162 to 0.568 (Mn), 16.963 to 37.366 (Fe), 0.0006 to 0.0074 (Co), 0.014 to 0.127 (Ni), 0.434 to 0.821 (Cu), 4.611 to 8.505 (Zn), 0.007 to 0.033 (As), 0.051 to 0.339 (Se), 0.0006 to 0.0021 (Cd), 0.006 to 0.112 (Pb). These were significantly lower contents than the PTDI for either Cr, which was 3 μg/kg/body weight per day, Mn, which was 140 μg/kg bw/day, Fe, which was 800 μg/kg bw/day, Co, which was 500 μg/kg bw/day; Ni, which was 5 μg/kg bw/day; Cu, which was 500 μg/kg bw/day; Zn, which was 1000 μg/kg bw/day; As, which was 2.14 μg/kg bw/day; Se, which was 5 μg/kg bw/day; Cd, which was 0.8 μg/kg bw/day, and Pb, which was 3.57 μg/kg bw/day, respectively. In relation to the non-carcinogenic risk factor of heavy metals, the parameters (THQ and HI) were calculated using Formulae (2) and (3). The values of these parameters are indicated in Table 5.

If THQ was equal to, or lower than, 1, its adverse effect was considered insignificant; however, when the THQ was higher than 1, there was deemed to be a considerable non-carcinogenic health risk [27]. In this study, Cr, Mn, Fe, Co, Ni, Cu, Zn, As, Se, Cd and Pb in the THQ value of investigated egg samples ranged between 0.175 and 0.393 (Cr), 0.001 and 0.004 (Mn), 0.024 and 0.053 (Fe), 0.002 and 0.025 (Co), 0.001 and 0.006 (Ni), 0.011 and 0.021 (Cu), 0.015 and 0.028 (Zn), 0.023 and 0.110 (As), 0.010 and 0.068 (Se), 0.001 and 0.002 (Cd), 0.002 and 0.028 (Pb), respectively. Accordingly, the THQ values of investigated elements in egg samples coming from different Romanian regions were in the following order: Cr > As > Fe > Se > Zn > Cu > Co > Pb > Ni > Cd > Mn. These values indicated the absence of any significant non-carcinogenic health risk due to the consumption of eggs. Besides this, the HI values calculated were also less than 1. These results showed no noticeable health risks for egg consumers from the investigated regions in Romania.

## 3. Materials and Methods

### 3.1. Samples Description

**Figure 4:**
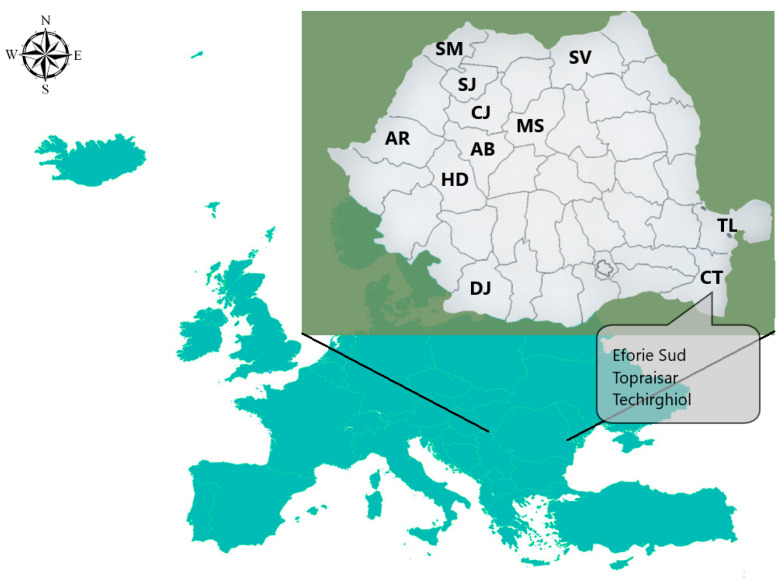
The map of the egg sample’s locations. AB, Alba County; AR, Arad County; CJ, Cluj County; CT, Constanta County; DJ, Dolj County; HD, Hunedoara County; MS, Mures County; SM, Satu Mare County; SJ, Salaj County; SV, Suceava County, TL, Tulcea County.

A total of 58 samples, consisting of egg yolk (n = 29) and egg albumen or egg white (n = 29) were investigated from the isotopic and elemental contents point of view. Of these, 28 samples were collected from different Romanian regions (e.g., Eforie Sud, Topraisar, Techirghiol, Constanta, Tulcea, Alba, Cluj, Satu Mare, Salaj, Mures, Suceava, Arad, Hunedoara, Dolj) (Figure 4) and one sample from Greece, coming from two different hen husbandry systems, namely: backyard (n = 15) and barn (n = 14, including the one of Greece). In the laboratory, before determining the isotopic and elemental profiles of the samples, each fresh egg was split up into its components, egg white, and yolk. Then, the water was extracted by a procedure that used cryogenic distillation under vacuum [28]. At the end of this procedure, the obtained egg white and yolk were completely dry, without any water content. In order to obtain the isotopic and elemental fingerprint of the eggs, each component (egg white and yolk) was prepared separately, according to a specific protocol.

### 3.2. Samples Preparation and Stable Isotopic Analysis

Each egg white sample (5 mg) was converted to CO_2_, by dry combustion (550 °C) for 3 h in oxygen excess. The obtained CO_2_ was purified from other combustion gases by cryogenic separation and subsequently measured by the Isotope Ratio Mass Spectrometry (IRMS) technique.

Lipids were extracted from the yolk due to the fact that fractions having high fat content were relatively depleted in ^13^C versus low-lipid fractions [29]. For lipids removal, the yolk was homogenized using a pestle and mortar. Then, a mix of chloroform and methanol (1:2, *v*/*v*) was used in the process of sample preparation. The resulting delipidated yolk was dried in an oven at 55 °C, 48 h, before the dry combustion stage, for future isotopic analysis.

The isotopic fingerprint of ^13^C was determined using an isotope ratio mass spectrometer (Delta V Advantage, Thermo Scientific, Waltham, MA, USA) in line with a dual inlet system. The components of egg samples were measured in duplicate. Each day, one working standard was measured before starting sample analyses. This working standard was calibrated against NBS–22 oil certified reference material from IAEA Vienna (International Atomic Energy Agency), which had an isotopic composition of δ^13^C_VPDB_ = −30.03‰. The uncertainty was ±0.3‰.

The ^13^C isotopic composition (fingerprint or signature) was reported in conventional δ notation versus international standard Vienna Pee Dee Belemnite (V-PDB), according to Equation (1) [30]:

(1) δXi=RsampleRstandard-1∗1000

where *i* represents the mass number of the heavier isotope of the element *X* (^13^C, ^2^H, ^18^O), *R_sample_* is the isotope number ratio of a sample (^13^C/^12^C; ^2^H/^1^H; ^18^O/^16^O), and *R_standard_* is that of the international standard. The delta values were multiplied by 1000 and expressed in units “per mil” (‰).

### 3.3. Samples Digestion Procedure and Elemental Analysis

The dry edible parts of the egg (egg white and yolk) were ground by means of an agitating mortar to obtain a fine powder. In order to determine the elemental content by ICP–MS analysis, a microwave digestion procedure was applied. Quantities of 0.1 g of samples were accurately weighed in a PTFE digestion vessel, and then 4 mL of HNO_3_ (60% *v*/*v*) and 1 mL of H_2_O_2_ (30% *v*/*v*) were added for sample mineralization, using a microwave digester (Speed ENTRY by Berghof^®^). The microwave system was set to ramp from room temperature to 80 °C in 3 min, held for 5 min, and then from 80 °C to 130 °C in 5 min, kept for 10 min, and after this, to 190 °C in 5 min, held for 15 min, and, finally, from 190 °C to 75 °C in 5 min, and held for 10 min. The digested solutions were left to cool at room temperature, then diluted with ultrapure water (resistivity 18 MΩ cm^−1^, Millipore, Bedford, MA, USA water purification system) to a final volume of 50 mL. The elemental concentrations were analyzed by ICP–MS, using an ELAN RDC (e) mass spectrometer (PerkinElmer SCIEX^®^, USA) equipped with a Meinhart nebulizer. The operating conditions were as follows: nebulizer gas flow rates—0.92 L/min; auxiliary gas flow—1.2 L/min; plasma gas flow—15 L/min; lens voltage—7.25 V; radiofrequency power—1100 W; CeO/Ce—0.020; Ba++/Ba+—0.015. Certified multi-element solutions (10 μg/mL and 10 mg/L, respectively, PerkinElmer Pure Plus, U.S.A.) were used for the preparation of the standard stock solutions, by dissolving the multi-element solutions with ultrapure water. For the calibration curve, the working solutions of specific concentration and volume were prepared by diluting the stock solution. Since there was no matching certified reference material for eggs, the method’s accuracy was checked by using NCS ZC85006 as the standard reference material.

### 3.4. Data Analysis

The building of the Partial Least Squares Discriminant Analysis (PLS–DA) models used the SOLO 8.9.1, 2021 (Eigenvector Research Incorporated, USA) software, based on the isotopic and elemental fingerprint of egg components. The PLS–DA models were developed in order to differentiate the egg constituents from two hen rearing systems (backyard and barn). Scatter plots of scores from the latent variables (LVs) were used to study the distribution of samples. PLS-DA was applied for the identification of the most important variables (parameters), based on which the classification model was achieved. The performance of models was evaluated by applying the cross-validation technique and by computing the accuracy, sensitivity and specificity measures.

### 3.5. Human Health Risk Assessment and Estimation of Non-Carcinogenic Risk

The human non-carcinogenic health risk was evaluated based on the following health risk requirements, namely, the daily intake (EDI) and the target hazard quotient (THQ), according to the suggested model by the US EPA (United State Environment Protection Agency) [25,31].

#### 3.5.1. Estimated Daily Intake (EDI) of Heavy Metals through Eggs Consumption

The daily intake of metals is a fundamental parameter for health risk assessments and depends on three significant factors, namely, the concentration of metals in food (eggs, in our case), the daily consumption of food items, as well as the body weight of human beings [32]. The EDI values of heavy metals for consumers were calculated by Equation (2) [31,33]:

EDI = (C × IR_d_)/BW (2)

where C is the concentration of elements in egg samples (mg/kg wet weight), IR_d_ represents the daily ingestion rate (g/day) for the Romanian population (40 g) [4] and BW is the body weight (kg) (70 kg for an adult).

#### 3.5.2. Estimation of Non-Carcinogenic Risks

The potential non-carcinogenic effect of elements was determined using THQ by means of Equation (3) [32]:

THQ = EDI/R_f_ D (3)

where EDI dose (mg/kg body weight per day) of the heavy metals and R_f_ D is an oral reference dose of the elements that have no harmful effect during a lifetime (mg/kg/day).

If THQ <1, it meant that the exposed population was assumed to be safe [34]. The total THQ (TTHQ) or hazard index (HI) of elements for the eggs was estimated because people suffer combined effects from exposure to several contaminants [32]. The TTHQ was calculated using Equation (4) [35]:

TTHQ = HI = THQ (element 1) + THQ (element 2) + THQ (element 3) +… + THQ (element n)(4)

## 4. Conclusions

In the present study, the isotopic and elemental signatures of 58 samples (29 egg white and 29 egg yolk) were assessed. In order to differentiate the eggs coming from hens reared in the backyard rearing system from those originating from hens reared in the barn system, differentiation models based on PLS–DA were developed. Building on the most significant features identified, the PLS–DA led to a total accuracy score of 96% for egg white classification according to the hen’s rearing system. Regarding yolk samples, based on the most important differentiation markers, a percentage of 100% was obtained.

Another objective of our work was related to the human health risk assessment and estimation of non-carcinogenic risk. Our results showed that the EDI levels of Cr, Mn, Fe, Co, Ni, Cu, Zn, Se, As, Cd and Pb were significantly lower than the PTDI values established by FAO/WHO. The non-carcinogenic risk values for eating eggs coming from the study area were in the safe range for consumers, indicating no health risks by consumption of this foodstuff.

## Figures and Tables

**Table 1:** The minimum (min) and maximum (max) values of elements having the highest classification power according to hen rearing system.

Element	Egg White				Egg Yolk			
	Backyard		Barn		Backyard		Barn	
	Value							
	min	max	min	max	min	max	min	max
**δ^13^C (‰)**	−25.1	−14.0	−21.8	−15.9	−25.3	−14.4	−23.7	−15.9
Li (mg/kg) *	0.02	2.37	0.002	0.03	0.01	0.50	0.001	0.04
B (mg/kg) *	0.07	0.74	0.17	0.43	0.002	0.42	0.08	0.67
Mg (mg/kg) *	67.2	171.4	56.9	164.0	128.9	233.9	110.7	214.8
K (mg/kg) *	492.5	2010.8	424.7	1211.3	912.3	1634.3	752.7	1298.8
Ca (mg/kg) *	18.0	209.0	17.4	69.0	888.3	1704.9	963.8	1803.0
Mn (mg/kg) *	0.01	0.07	0.01	0.02	0.53	4.20	0.60	1.96
Fe (mg/kg) *	0.66	9.82	0.30	1.93	74.57	148.75	72.10	112.52
Co (μg/kg) *	0.12	4.70	0.40	2.47	6.94	36.28	2.40	12.11
Zn (mg/kg) *	0.002	1.05	0.03	0.21	18.85	46.32	19.95	36.07
Rb (mg/kg) *	0.26	1.56	0.58	1.90	0.38	1.23	0.69	1.64
Sr (mg/kg) *	0.06	0.36	0.02	0.10	0.78	3.10	0.37	0.74
Mo (μg /kg) *	0.29	23.00	4.36	13.97	6.07	128.04	69.70	246.40
Ba (mg/kg) *	0.01	0.10	0.003	0.06	0.96	17.79	0.40	3.42
La (μg /kg) *	0.03	1.79	0.02	0.41	0.20	1.44	0.19	8.50
Ce (μg /kg) *	0.03	1.81	0.02	0.57	0.20	3.78	0.17	5.39
Pb (mg/kg) *	0.001	0.59	0.002	0.06	0.03	0.17	0.02	0.06

* elemental concentration is expressed in units of fresh weight.

**Table 2:** The mean concentration (in mg/kg fresh weigh) of all investigated elements by ICP-MS.

**Sample Type**	**Growing System**	**Na**	**Mg**	**K**	**Ca**	**Li**	**B**	**Sc**	**Ti**	**V**	**Cr**	**Mn**	**Fe**	**Ni**	**Cu**	**Zn**	**Se**	**Rb**	**Sr**
white	backyard	1748.27	108.35	838.00	49.67	0.31	0.24	0.02	0.22	0.09	0.35	0.02	2.98	0.03	0.19	0.18	0.10	0.60	0.16
	barn	1812.17	116.23	798.19	42.40	0.01	0.30	0.02	0.23	0.09	0.42	0.01	1.36	0.03	0.20	0.08	0.10	1.01	0.06
yolk	backyard	887.12	180.08	1292.95	1350.70	0.11	0.22	0.22	1.50	0.32	3.28	1.34	114.27	0.28	2.40	30.67	0.60	0.75	1.70
	barn	830.47	164.24	1028.51	1364.54	0.01	0.33	0.19	1.00	0.30	3.16	1.21	94.06	0.28	2.35	28.00	0.56	1.06	0.61
**Sample type**	**growing system**	**Pd**	**Ba**	**Co**	**Zr**	**Nb**	**Mo**	**Ag**	**La**	**Ce**	**Pr**	**Gd**	**Pt**	**As**	**Cd**	**Sn**	**Sb**	**Pb**	
white	backyard	0.03	0.03	0.001	0.005	0.0003	0.005	0.01	0.0002	0.0003	0.0002	0.10	0.005	0.02	0.001	0.03	0.0005	0.05	
	barn	0.03	0.01	0.001	0.01	0.0003	0.01	0.01	0.0001	0.0001	0.0003	0.04	0.005	0.03	0.001	0.04	0.0004	0.01	
yolk	backyard	0.23	5.22	0.017	0.05	0.001	0.08	0.07	0.0006	0.0009	0.0010	0.13	0.02	0.05	0.005	0.23	0.0023	0.06	
	barn	0.22	1.13	0.008	0.07	0.001	0.12	0.04	0.0016	0.0007	0.0006	0.10	0.03	0.05	0.006	0.31	0.0020	0.03	

**Table 3:** The essential trace elements and toxic metals concentrations (mg/kg fresh weight) of whole egg samples.

Sample Code	Origin	Cr	Mn	Fe	Co	Ni	Cu	Zn	Se	Cd	Pb	As
**Egg-1**	Tulcea	1.02	0.30	40.23	0.003	0.06	0.73	8.11	0.19	0.003	0.03	0.02
**Egg-2**	Tulcea	1.86	0.37	41.64	0.005	0.14	1.08	13.35	0.30	0.004	0.03	0.02
**Egg-3**	Constanta	1.22	0.22	31.50	0.004	0.10	1.00	7.68	0.14	0.004	0.37	0.02
**Egg-4**	Constanta	1.56	0.45	45.02	0.008	0.10	1.04	11.22	0.33	0.004	0.03	0.03
**Egg-5**	Eforie Sud	1.57	0.50	49.01	0.006	0.16	1.09	11.49	0.16	0.003	0.07	0.03
**Egg-6**	Topraisar	2.06	0.60	65.39	0.013	0.22	1.44	14.74	0.55	0.004	0.05	0.06
**Egg-7**	Techirghiol	1.62	0.38	56.01	0.007	0.06	1.31	11.44	0.23	0.003	0.03	0.03
**Egg-8**	Alba	1.93	0.51	53.26	0.009	0.20	1.17	13.26	0.43	0.001	0.03	0.02
**Egg-9**	Alba	1.50	0.51	50.99	0.005	0.14	1.11	11.87	0.39	0.003	0.02	0.03
**Egg-10**	Cluj	1.36	0.28	32.48	0.006	0.11	1.13	11.23	0.59	0.003	0.02	0.04
**Egg-11**	Salaj	1.97	1.69	53.00	0.015	0.17	1.27	16.00	0.31	0.001	0.02	0.05
**Egg-12**	Salaj	1.65	0.37	54.80	0.008	0.09	1.04	13.77	0.36	0.003	0.03	0.04
**Egg-13**	Salaj	0.76	0.92	53.78	0.013	0.23	0.98	18.67	0.14	0.001	0.03	0.04
**Egg-14**	Mures	1.46	0.37	34.23	0.006	0.15	0.76	10.23	0.31	0.003	0.01	0.02
**Egg-15**	Suceava	1.27	0.74	51.14	0.005	0.05	0.93	12.58	0.09	0.001	0.02	0.05
**Egg-16**	Satu Mare	1.51	0.79	39.75	0.004	0.19	1.10	11.98	0.29	0.003	0.01	0.03
**Egg-17**	Satu Mare	1.70	0.74	42.62	0.003	0.15	1.36	13.90	0.30	0.004	0.02	0.02
**Egg-18**	Satu Mare	1.54	0.77	42.50	0.003	0.13	1.38	14.45	0.29	0.003	0.01	0.03
**Egg-19**	Arad	1.36	0.58	38.63	0.004	0.10	0.91	11.25	0.33	0.003	0.01	0.03
**Egg-20**	Arad	1.57	0.59	46.04	0.004	0.07	1.12	12.76	0.17	0.003	0.01	0.03
**Egg-21**	Arad	1.27	0.75	33.19	0.005	0.09	0.89	11.27	0.15	0.003	0.01	0.05
**Egg-22**	Hunedoara	1.50	0.24	34.31	0.002	0.10	0.86	9.19	0.33	0.003	0.01	0.04
**Egg-23**	Hunedoara	1.60	0.36	36.06	0.004	0.14	1.05	10.73	0.39	0.003	0.03	0.05
**Egg-24**	Hunedoara	1.79	0.32	40.75	0.006	0.16	1.21	11.02	0.37	0.004	0.01	0.05
**Egg-25**	Hunedoara	1.65	0.35	36.81	0.003	0.20	1.14	12.06	0.26	0.003	0.01	0.04
**Egg-26**	Hunedoara	1.67	0.29	38.67	0.003	0.13	1.08	10.12	0.13	0.003	0.01	0.04
**Egg-27**	Hunedoara	1.46	0.26	34.22	0.003	0.15	0.91	8.05	0.29	0.004	0.05	0.04
**Egg-28**	Dolj	1.66	0.50	44.89	0.003	0.14	0.95	12.60	0.42	0.003	0.01	0.03
**Egg-29**	Greece	0.92	0.33	29.69	0.001	0.02	0.86	8.07	0.24	0.002	0.01	0.01
**R**_**f**_ D *		0.003	0.14	0.7	0.0003	0.02	0.04	0.3	0.005	0.001	0.004	0.0003

* R_f_D (mg/kg/day)—oral reference dose established by US Environmental Protection Agency (USEPA) [25].

**Table 4:** Estimated dietary intakes (EDI) of investigated elements by consumption of eggs in different regions and provisional tolerable daily intake (PTDI).

No.	Origin	EDI (μg/kg/Body Weight/Day)										
		Cr	Mn	Fe	Co	Ni	Cu	Zn	Se	As	Cd	Pb
1	Tulcea (n = 2)	0.822	0.192	23.390	0.0024	0.056	0.516	6.131	0.141	0.011	0.0019	0.016
2	Constanta (n = 2)	0.796	0.191	21.865	0.0033	0.056	0.581	5.400	0.133	0.014	0.0021	0.112
3	Eforie Sud (n = 1)	0.900	0.285	28.003	0.0032	0.089	0.624	6.565	0.093	0.017	0.0016	0.041
4	Topraisar (n = 1)	1.178	0.344	37.366	0.0074	0.127	0.821	8.425	0.312	0.033	0.0020	0.027
5	Techirghiol (n = 1)	0.927	0.217	32.007	0.0040	0.036	0.746	6.538	0.130	0.020	0.0020	0.016
6	Satu Mare (n = 3)	0.905	0.436	23.787	0.0020	0.097	0.703	7.396	0.169	0.014	0.0017	0.008
7	Arad (n = 3)	0.800	0.365	22.449	0.0023	0.048	0.581	6.860	0.142	0.018	0.0017	0.008
8	Hunedoara (n = 6)	0.921	0.173	21.031	0.0017	0.068	0.545	5.691	0.205	0.026	0.0019	0.012
9	Alba (n = 2)	0.981	0.292	29.786	0.0038	0.098	0.650	7.179	0.233	0.016	0.0015	0.013
10	Cluj (n = 1)	0.776	0.162	18.560	0.0032	0.064	0.647	7.179	0.339	0.025	0.0011	0.014
11	Salaj (n = 3)	0.835	0.568	30.778	0.0067	0.073	0.660	8.505	0.191	0.027	0.0016	0.015
12	Mures (n = 1)	0.832	0.214	19.562	0.0032	0.083	0.434	5.843	0.175	0.010	0.0019	0.007
13	Dolj (n = 1)	0.948	0.285	25.652	0.0017	0.082	0.541	7.201	0.238	0.018	0.0006	0.006
14	Suceava (n = 1)	0.726	0.422	29.222	0.0030	0.030	0.530	7.189	0.051	0.031	0.0014	0.013
15	Greece (n = 1)	0.526	0.186	16.963	0.0006	0.014	0.489	4.611	0.134	0.007	0.001	0.008
PTDI		3	140	800	500	5	500	1000	5	2.14	0.8	3.57

n represents the number of investigated samples. Provisional tolerable daily intake value (in μg/kg/body weight per day) of metals established by the Joint FAO/WHO Expert Committee on Food Additives (JECFA, 2011) [26].

**Table 5:** Target hazard quotient (THQ) and non-carcinogenic (HI) risk from egg consumption from the study area.

No.	Origin	THQ of Individual Elements											∑THQ
		Cr	Mn	Fe	Co	Ni	Cu	Zn	As	Se	Cd	Pb	HI
1	Tulcea (n = 2)	0.274	0.001	0.033	0.008	0.003	0.013	0.020	0.036	0.028	0.002	0.004	0.423
2	Constanta (n = 2)	0.265	0.001	0.031	0.011	0.003	0.015	0.018	0.045	0.027	0.002	0.028	0.446
3	Eforie Sud (n = 1)	0.300	0.002	0.040	0.011	0.004	0.016	0.022	0.058	0.019	0.002	0.010	0.483
4	Topraisar (n = 1)	0.393	0.002	0.053	0.025	0.006	0.021	0.028	0.110	0.062	0.002	0.007	0.709
5	Techirghiol (n = 1)	0.309	0.002	0.046	0.013	0.002	0.019	0.022	0.066	0.026	0.002	0.004	0.510
6	Satu Mare (n = 3)	0.302	0.003	0.034	0.007	0.005	0.018	0.025	0.045	0.034	0.002	0.002	0.476
7	Arad (n = 3)	0.267	0.003	0.032	0.008	0.002	0.015	0.023	0.059	0.028	0.002	0.002	0.440
8	Hunedoara (n = 6)	0.307	0.001	0.030	0.006	0.003	0.014	0.019	0.085	0.041	0.002	0.003	0.511
9	Alba (n = 2)	0.327	0.002	0.043	0.013	0.005	0.016	0.024	0.053	0.047	0.002	0.003	0.534
10	Cluj (n = 1)	0.259	0.001	0.027	0.011	0.003	0.016	0.024	0.085	0.068	0.002	0.003	0.498
11	Salaj (n = 3)	0.278	0.004	0.044	0.022	0.004	0.017	0.028	0.089	0.038	0.001	0.004	0.529
12	Mures (n = 1)	0.277	0.002	0.028	0.011	0.004	0.011	0.019	0.032	0.035	0.002	0.002	0.423
13	Dolj (n = 1)	0.316	0.002	0.037	0.006	0.004	0.014	0.024	0.059	0.048	0.002	0.002	0.512
14	Suceava (n = 1)	0.242	0.003	0.042	0.010	0.001	0.013	0.024	0.102	0.010	0.001	0.003	0.451
15	Greece (n = 1)	0.175	0.001	0.024	0.002	0.001	0.012	0.015	0.023	0.027	0.001	0.002	0.285
R_f_D values (mg kg^−1^ day^−1^)		0.003	0.14	0.7	0.0003	0.02	0.04	0.3	0.0003	0.005	0.001	0.004	

## Data Availability

Not applicable.

## Sample Availability

Samples are not available from the authors.
